# More than microglia: myeloid cells and biomarkers in neurodegeneration

**DOI:** 10.3389/fnins.2024.1499458

**Published:** 2024-10-31

**Authors:** Eleftheria Kodosaki, Rosie Bell, Aitana Sogorb-Esteve, Katharine Wiltshire, Henrik Zetterberg, Amanda Heslegrave

**Affiliations:** ^1^Department of Neurodegenerative Disease, UCL Institute of Neurology, London, United Kingdom; ^2^UK Dementia Research Institute at UCL, London, United Kingdom; ^3^Dementia Research Centre, UCL Queen Square Institute of Neurology, University College London, London, United Kingdom; ^4^Department of Psychiatry and Neurochemistry, Institute of Neuroscience and Physiology, The Sahlgrenska Academy at the University of Gothenburg, Mölndal, Sweden; ^5^Clinical Neurochemistry Laboratory, Sahlgrenska University Hospital, Mölndal, Sweden; ^6^Hong Kong Center for Neurodegenerative Diseases, Clear Water Bay, Hong Kong SAR, China; ^7^Wisconsin Alzheimer’s Disease Research Center, University of Wisconsin School of Medicine and Public Health, University of Wisconsin-Madison, Madison, WI, United States

**Keywords:** myeloid biomarkers, neuroinflammation, neurodegeneration, myeloid cells, microglia, biomarker utility, biomarker standardization

## Abstract

The role of myeloid cells (granulocytes and monocytes) in neurodegeneration and neurodegenerative disorders (NDD) is indisputable. Here we discuss the roles of myeloid cells in neurodegenerative diseases, and the recent advances in biofluid and imaging myeloid biomarker research with a focus on methods that can be used in the clinic. For this review, evidence from three neurodegenerative diseases will be included, Alzheimer’s disease (AD), Parkinson’s disease (PD), and multiple sclerosis (MS). We discuss the potential for these biomarkers to be used in humans with suspected NDD as prognostic, diagnostic, or monitoring tools, identify knowledge gaps in literature, and propose potential approaches to further elucidate the role of myeloid cells in neurodegeneration and better utilize myeloid biomarkers in the understanding and treatment of NDD.

## 1 Introduction

Myeloid cells are classically defined as granulocyte (e.g., neutrophils, mast cells) and monocyte/dendritic (monocytes, macrophages, dendritic cells)-lineage cells originally derived from the bone marrow (De Kleer et al., 2014). However, myeloid stem cells also develop into erythrocytes and platelets (Svoboda and Bartunek, 2015). An interesting exception is the main myeloid cell type in the brain, the microglia that arise from primitive yolk sac macrophages that engraft the developing neuroectoderm destined to become the central nervous system (CNS) parenchyma during embryogenesis (Mendes and Majewska, 2021; Bennett et al., 2018). However, other CNS-resident macrophages have been identified with different origins and characteristics (Prinz et al., 2011; Dermitzakis et al., 2023), and also other myeloid cells [i.e., dendritic cells, granulocytes, such as resident neutrophils and mast cells (Silver et al., 1996), and monocytes (once these infiltrate)]. Although not in the CNS, other peripheral myeloid cells have also been shown to be involved in NDD, including granulocytes, platelets, and erythrocytes, as well as peripheral tissue macrophages (Gopinath et al., 2020). As the brain is surrounded by cerebrospinal fluid (CSF) and communicates with the periphery via the blood–brain barrier (BBB), biomarker analysis focusing on CSF and blood to detect biomarkers from myeloid cells that indicate their status, such as activation or senescence, are extremely useful in studying these cells with respect to their contributions and behavior in NDD. These biomarkers are usually investigated using immunoassays or mass spectrometry methods, to detect quantities and the different forms of proteins. In addition, in the CSF and blood the cells themselves can be used as biomarkers either in their behavior (e.g., how they respond to stimuli), surface expression markers, epigenetic markers, or disease-related myeloid specific genetic polymorphisms. Imaging biomarkers for myeloid cells have also been explored. This review will briefly focus on the myeloid cells and biomarkers in AD, PD, and MS, as they are major NDD of different etiology with both shared and distinct progression mechanisms.

## 2 Myeloid cells and their role in neurodegeneration

### 2.1 Microglia and monocyte/dendritic lineage (monocytes, macrophages, dendritic cells)

As the brain’s resident immune cells, microglia play a variety of roles in the development and manifestation of NDD. They are found throughout the brain and exhibit both heterogeneity and plasticity, suggesting that they are a versatile cell type responding to different exposures (Benusa et al., 2020; Augusto-Oliveira et al., 2022). They not only reside in the brain tissue; evidence also shows their presence in the CSF (Esaulova et al., 2020), suggesting that the ability to travel through the CNS. The other resident CNS macrophages are restricted to specific locations (e.g., border-associated macrophages) and have roles correlating to these areas; however, they also exhibit heterogeneity in both their characteristics and functions (Dermitzakis et al., 2023; Sun and Jiang, 2024; Mildenberger et al., 2022). CNS macrophages are involved brain homeostasis and are the cells that are often the first line of response to conditions such as injury or infection. That response includes the secretion of molecules ranging from proteins to inflammatory mediators and vesicles (Kremlev et al., 2004; Lee et al., 2002; Paolicelli et al., 2019; Gibbons and Dragunow, 2006), alteration of membrane receptor expression levels and variability to control damage by phagocytosis and communication with other cells to elicit protective tissue responses (Fu et al., 2014); these roles are often dysregulated in NDD and aging with consequences such as accumulation of misfolded proteins which can lead to neurotoxicity and neurodegeneration (Gabandé-Rodríguez et al., 2020; Nizami et al., 2019). Both overactivation and exhaustion of myeloid cells in the CNS have been suggested to contribute to NDD.

Non-microglia CNS resident macrophages are often found in the meninges, perivascular spaces, and choroid plexus (Mildenberger et al., 2022). They take part in microglial-like functions, such as immune surveillance and maintenance of homeostasis, but they also have further roles in regulating CNS entry via modulation of the BBB of, e.g., circulating peripheral cells and molecules (Dermitzakis et al., 2023; Giladi et al., 2020), they are also involved in the glymphatic system, the dysfunction of which has been linked to accumulation of NDD-related proteins such as amyloid-*β* and tau in the brain (Da Mesquita and Rua, 2024).

Non-resident CNS macrophages may also enter the brain from the periphery as monocytes migrating across the blood–brain barrier under certain conditions such as disease. In the brain tissue they may subsequently differentiate into microglia-like cells, often expressing microglia cell-based markers, and affect NDD processes (Silvin et al., 2023). However, these cells do not become permanent residents of the CNS (De Vlaminck et al., 2022), and have been shown to have different functions to resident immune cells with some studies indicating anti-inflammatory and regulatory functions (De Vlaminck et al., 2022; Greenhalgh et al., 2018; Shechter et al., 2009), while damaging actions in the CNS have also been shown (DePaula-Silva, 2024; Fiala et al., 2002). Peripheral macrophages and monocytes [with their ability to engulf amyloid (Hallé et al., 2015)] and peripheral nervous system macrophages [with the latter overlapping in activation and homeostatic gene expression with microglia (Wang et al., 2020)], have been implicated to be involved in neurodegenerative processes in the CNS (Wakabayashi et al., 2010; Yang et al., 2020), either via their interactions with the BBB, disease-specific factors, inflammatory processes, or via crosstalk with other CNS cells (Yu et al., 2022).

Dendritic cells (DCs) are mainly regulatory antigen-presenting cells of the peripheral immune system, primarily known for their role in initiating and regulating immune responses. Recent studies, mainly in mice have suggested the existence of cells with DC characteristics in the CNS, especially where border-associated macrophages reside (D’Agostino et al., 2012). They are optimally positioned to interact with and activate T cells, which can make them accidental culprits for targeting of other CNS cells by T cells, which may contribute to neurodegeneration or neuroinflammatory injury in diseases such as MS (D’Agostino et al., 2012). They are also able to produce cytokines and recruit more immune cells (including more DCs) to the CNS and therefore exacerbate neuroinflammation and BBB disruption, although neuroprotective roles have also been attributed to them (Gallizioli et al., 2020; Ludewig et al., 2016; Pashenkov et al., 2003).

### 2.2 Granulocytes

One of the main type of granulocytes under investigation are neutrophils, which under normal conditions are found in the CNS in very low numbers in the meninges, pia membrane, and the CSF (Kanashiro et al., 2020). They can infiltrate the CNS via a compromised BBB. There they contribute to neuroinflammation and neurodegeneration with the generation of inflammatory molecules in AD (Jacobs et al., 2024), MS where a primed phenotype has been found (Naegele et al., 2012), and PD where their infiltration differs between brain areas and correlates with neuronal damage (Ji et al., 2008). Clinically, dramatically increased CSF neutrophil count is pathognomonic for bacterial meningitis, which induces prominent recruitment of neutrophils to the CNS within minutes of the infection (Fuchs et al., 2019). Other types of granulocytes are under-investigated in NDD. Eosinophils, cells involved in the development of allergic reactions and infection, contribute to the overall inflammatory environment that is often found in NDD periphery and have been shown to have neurotoxic effects upon entry in the CNS with disease-specific findings (Weaver et al., 1988; Inoue et al., 1996). Similar to eosinophils, basophils are involved in the recruitment of cells that potentially could be responsible for certain symptoms that arise during NDD (Miyake et al., 2022). Lastly, mast cells have also been shown to increase neuroinflammation in neurodegeneration when they release neuroinflammatory mediators, and have been observed in the CNS under physiological and pathological conditions (Silver et al., 1996; Duraisamy et al., 2019).

### 2.3 Erythrocytes and platelets

Erythrocytes are not involved *per se* in the development of NDD, but alterations in their function have been shown during the course of NDD, so there could be a link with disease progression. As they are responsible for oxygen delivery and have a significant role in oxidative stress, issues arising with their function may lead to problems with the energy required for brain metabolic processes; indeed issues with energy and metabolism have been found throughout the spectrum of NDD (Kosenko et al., 2020; Park and Choi, 2020; Johansson et al., 2020) and a case has been made for their role in iron accumulation which has also been related to NDD processes (Prohaska et al., 2012). Haemolysis of erythrocytes in the brain parenchyma after micro- or macrobleeds or upon traumatic brain injury may be causally related to the development of tau pathology in diseases like superficial CNS siderosis (Kondziella and Zetterberg, 2008) and chronic traumatic encephalopathy (Juan et al., 2022).

Platelets may play a role in the development and progression of NDD, with the prime example being AD. Blood platelets produce amyloid beta (Aβ) peptides, using the same machinery as neurons and oligodendrocytes [the main Aβ producers in the brain (Rajani et al., 2024; Sasmita et al., 2024; Plant et al., 2003; Hampel et al., 2021)], and the precursor protein of Aβ, amyloid precursor protein (APP), was soon after cloning (Kang et al., 1987; Goldgaber et al., 1987) identified as the previously described coagulation factor protease nexin-II (Nostrand et al., 1989; Oltersdorf et al., 1989). However, whether blood platelets contribute to Aβ plaque formation in the brain remains unknown (Humpel, 2017). Nevertheless, they have been found to contribute to the vascular pathology of AD (Zhang et al., 2013). In MS, there is evidence for platelet-leukocyte interactions, which contribute to the pathophysiology of the disease (Dziedzic and Bijak, 2019), and potentially may be relevant in PD as well (Beura et al., 2022). For both erythrocytes and platelets, changes in their function or morphology may be considered as biomarkers in NDD, discussed further below.

## 3 Myeloid biomarkers

The term biomarker is defined by the National Institutes of Health Biomarkers Definitions Working Group as “a characteristic that is objectively measured and evaluated as an indicator of normal biological processes, pathogenic processes, or pharmacologic responses to a therapeutic intervention” (Biomarkers Definitions Working Group et al., 2001). Myeloid-related molecules such as proteins (and variations thereof) found in blood and CSF are quantified by immunoassays or mass spectrometry, indicate disease-or disease processes and are therefore considered myeloid biomarkers. Other biomarkers are myeloid cell characteristics (activation/function, number) measured by imaging, and variations of the different myeloid cells found in biofluids and tissues.

A rather gray area is the field of genomic/genetic biomarkers, as genotypes are not biomarkers *per se* due to them not reflecting biological or pathological processes; however, they can be associated with biomarkers. Epigenetic changes, on the other hand can be considered biomarkers, and so can the results of these changes. In this section, we will first discuss findings of typical biomarkers of NDD found in biofluids that can be measured with, e.g., immunoassays. We will then move to those less commonly thought of as biomarkers (including the gray area genetics-related myeloid biomarkers), and lastly, we will discuss findings from imaging myeloid cell biomarkers studies.

### 3.1 Biofluid

Triggering receptor expressed on myeloid cells 2 (TREM2), is expressed in microglia and indicates activation, but also in other CNS and non-CNS macrophages, monocytes, dendritic cells, and granulocytes (Jay et al., 2017), is one of the most studied myeloid NDD biomarkers. A major reason for this focus is the identification of a loss of function mutation in the *TREM2* gene as a genetic etiology of AD (Guerreiro et al., 2013; Jonsson et al., 2013). Its soluble form (sTREM2) can be measured in both CSF and blood, and elevated levels have been associated with AD in a variety of CSF (Piccio et al., 2016; Heslegrave et al., 2016) and plasma (Hu et al., 2014; Zhao et al., 2022) studies, hinting that the function of TREM2 is protective rather than detrimental for AD (Brown and St George-Hyslop, 2022; Nabizadeh et al., 2024). Nevertheless, the plasma findings regarding sTREM2 have not been confirmed (Ashton et al., 2019), and some data suggest that plasma sTREM2 concentration is likely linked to cerebrovascular dysfunction rather than AD (Tsai et al., 2021). In other NDD such as PD there are also some findings for sTREM2, where it was found to potentially indicate indirectly toward neuronal injury (Wilson et al., 2020) whereas elsewhere higher CSF sTREM2 predicted a more rapid cognitive decline (Qin et al., 2022). In MS and other neuroinflammatory conditions, CSF sTREM2 concentration is increased (Piccio et al., 2008; Öhrfelt et al., 2016), and reduced after treatment (Öhrfelt et al., 2016). Another genetically linked biomarker is the presence of a specific isoform of Apolipoprotein E (APOE). The protein has three isoforms: E2, E3, E4 and most people express apoE E3. The protein is involved in lipid metabolism, been shown to affect the function of myeloid cells (Bonacina et al., 2018) [including via interacting with patient sex as a factor and affecting the interactions of microglia with plaques in mice (Stephen et al., 2019)] and is a ligand for TREM2 (Atagi et al., 2015). The AD genetic risk variant, *ApoE ɛ4* is considered an important genetically linked biomarker for AD pathophysiology as it correlates with enhanced amyloid plaque formation (Drzezga et al., 2009), and in humans it has been found associated with an increased immune response (Gale et al., 2014). *ApoE ε4* has been linked with differential biomarker profiles depending on the AD-related amyloid presence (Reinvang et al., 2013), whereas the APOE isoforms quantified in biofluids showed association with AD (Minta et al., 2020).

Amyloid, although due to its involvement in plaque formation is primarily considered a CNS biomarker as it is secreted by neurons, astrocytes, microglia [via extracellular vesicles (EVs)], and oligodendrocytes (Hampel et al., 2021; Trotta et al., 2018; Skaper et al., 2009). It is also secreted in the periphery primarily by platelets (Chen et al., 1995); unlike tau [which has also been found in microglia-derived EVs (Trotta et al., 2018) and has a distinct brain-derived form (Gonzalez-Ortiz et al., 2023)] so far there is no specific way to distinguish between brain and blood derived amyloid. Interestingly, though, various methods of amyloid detection do show some inconsistencies in the correlations between their measurements (Pannee et al., 2021). This could be attributed to assay-specific conditions and characteristics, or antibodies raised against epitopes which are not necessarily reflecting changes to the sequence of the proteins, but perhaps to structure. For example, the coagulation system has also been found to exhibit changes in its levels of certain biomarkers in NDD (Begic et al., 2020; Park et al., 2021; Festoff et al., 2016; Sharma et al., 2021; Bartl et al., 2023; Ziliotto et al., 2018; Koudriavtseva et al., 2023), and members of the coagulation system have been known to act as chaperones for amyloid and have been shown to interfere with its ability to aggregate (Geraghty et al., 2021) while others have been found to contribute toward neurodegeneration and amyloid aggregation (Ahn et al., 2017). Other chaperone proteins, such as Heat shock proteins (HSP)70/90 have also been found to be involved in NDD via their potential interactions with amyloid, and have been found to change with disease progression (Son et al., 2015). So, differences in amyloid between studies could be attributed -in part- to its differential interaction with chaperones and other proteins. Also expressed by platelets in the periphery, the chemokine platelet factor 4 (PF4) (Jian et al., 2017) has exhibited differences in blood levels in AD when compared to controls (Yang et al., 2023), and similarly for MS (Cananzi et al., 1987) and PD (Shen et al., 2020). Surprisingly though, PF4 was also found (in mice at least) to exhibit cognition enhancing effects (Schroer et al., 2023), and correlation with younger age in both mice and humans (Schroer et al., 2023), however the latter was not confirmed in another human study while cognition was not investigated in this study (Duchez et al., 2024).

Chitinase-3-like protein 1 (Ch3l1/YKL-40), an injury response protein that regulates tissue remodeling and is involved in inflammatory processes, is secreted by microglia as well as astrocytes in the CNS, and most of the peripheral myeloid cells (Llorens et al., 2017; Baldacci et al., 2017). It can be detected in both the blood and the CSF, and elevated levels have been observed in a variety of neurodegenerative and neuroinflammatory disorders (Llorens et al., 2017; Villar-Piqué et al., 2019). The structurally-similar chitotriosidase has also been studied and has been found to be a biomarker for microglial reactivity in the CNS (Olsson et al., 2012) and altered in NDD (Rosén et al., 2014; Møllgaard et al., 2016). Other markers include Monocyte Chemoattractant Protein-1 (MCP-1), which is detected both in CSF and blood and increased levels have been found in a variety of NDD where they are often related to disease progression, and CNS myeloid cell infiltration (Singh et al., 2021); this protein is secreted by myeloid cells, but also other CNS and BBB cells and attracts myeloid cells from the periphery to the CNS aiding in the movement of cells within the CNS (Yao and Tsirka, 2014). Interestingly, some biomarkers have altered relationships when it comes to their levels vs. controls depending on the disease, perhaps reflecting disease-specific neurodegenerative mechanisms; for example CX3CL1 is increased in the CSF and blood of people with both MS (Kastenbauer et al., 2003) and AD (Bivona et al., 2022) vs. controls, but decreased in PD (Hatcher-Martin et al., 2020), whereas it seems unaffected in non-AD dementia (Bivona et al., 2022), although in all the above mentioned conditions neurodegeneration is apparent. Similarly, sCD163 has been found increased in PD (Nissen et al., 2021) in CSF in women indicating a sex-specific disease activity present in blood. The same was also not true in CSF for the transition of patients with mild cognitive impairment (MCI) to AD for men but it is true for women (Gross et al., 2019). In MS a CSF/serum ratio (Stilund et al., 2015; Stilund et al., 2014) was linked to disease progression and was found increased vs. controls.

Other myeloid-related markers (that are mostly considered inflammatory/immune system activation markers) such as cytokines (Guerreiro et al., 2007; Kodosaki et al., 2024; Qu et al., 2023) and chemokines (including the aforementioned CX3CL1) have also attracted attention as potential biomarkers that can be secreted by the entire repertoire or majority of myeloid cells. In addition, polymorphisms in immune-related molecules such as cytokines have been found to play either protective or detrimental roles in the development of AD (Su et al., 2016) most of which can be attributed to their effects on cytokine production.

Complement system proteins and related proteins including lectin pathway proteins [such as galectin-3 (Boza-Serrano et al., 2022; Wang et al., 2015) and mannan-binding lectin (MBL) (Lanzrein et al., 1998)], as well as the more investigated classical and alternative pathways have exhibited differential expression in PD, AD, and MS (Kodosaki et al., 2024; Dufek et al., 2009; Naskar et al., 2022; Khosousi et al., 2023; Krance et al., 2021). Members of the complement system have also exhibited amyloid chaperone activity as well (Geraghty et al., 2021). Interestingly, a group of granulocyte specific activation markers have also been found as discriminatory between MS and related neurological disorders (Leppert et al., 2023). Similarly, neutrophil-related activation markers myeloperoxidase and neutrophil gelatinase-associated lipocalin were found increased in the peripheral blood of AD relative to controls (Wu et al., 2020) but most of these markers are also expressed in other myeloid and non-myeloid cells. Most markers mentioned here are not selective for one cell type or function; however, grouping biomarkers according to their function and cell type origin may offer advantages compared to focusing on single markers. Lastly, an under-investigated, but promising area of fluid biomarker research includes studying EVs depending on their cellular origin and content. There have been studies showing that certain microglial EVs may contribute to CNS processes such as neurogenesis via miRNA (Fan et al., 2022), and other studies have shown that the contents of EVs that relate to myeloid activity exhibit changes in AD (McKeever et al., 2018), MS (Palacio et al., 2023), and PD (Marchetti et al., 2020), however this is a developing field.

Another interesting area of research is using the cells themselves as biomarkers by employing techniques to evaluate cellular characteristics, for example flow cytometry for membrane markers, or microscopy for phenotypic changes, while epigenetic changes may also reveal novel biomarkers. Erythrocytes and platelets have both been shown to have alterations in functions, activation, and membrane characteristics in neurodegenerative disorders (Behari and Shrivastava, 2013; Bosman, 2018) with disease specific alterations found in platelets in AD (Davies et al., 1996), MS (Sheremata et al., 2008), and PD (Beura et al., 2022; Benecke et al., 1993; Factor et al., 1994). Dendritic cells have been shown by one study to be decreased in AD compared to controls, and associated with disease progression (Ciaramella et al., 2016). In this study, specific subsets of DC, namely myeloid DC were found depleted in the blood of AD patients however that was not observed when the patients were under AD treatment with acetylcholinesterase inhibitors (Ciaramella et al., 2016); elsewhere both subpopulations of DCs were found increased in both cognitively normal and patients with MCI who were amyloid positive (Grayson et al., 2023). In addition, monocyte-derived DC from AD patients exhibited more pro-inflammatory/reactive behavior via secretion of more inflammatory biomarkers following inflammatory stimuli exposure compared to controls (Ciaramella et al., 2010). For monocytes, differential expression of the surface markers CD14 and CD16 is correlated with the expression of other surface markers that can functionally categorize these cells (Savinetti et al., 2021); different categories are more prominent in the blood of patients with PD (Grozdanov et al., 2014), MS (Chuluundorj et al., 2014), and AD (Saresella et al., 2014), with the findings in AD also indicating a reduced amyloid uptake from these cells (Chen et al., 2020) which was not the case for PD patient derived cells in the same study. Similarly, in a relatively small study, patient derived monocytes showed differential activation and surface markers at baseline and upon inflammatory receptor [Toll-like receptor (TLR)-2 and TLR-4] activation, with inflammatory response peaking in the MCI stage compared to both controls, pre-MCI, and AD cells, and decreased phagocytic ability when moving from HC to the other categories (pre-MCI, MCI, AD) (Munawara et al., 2021). Overall, functional and numerical alterations in peripheral myeloid cells (including different populations such as granulocytes and monocyte-lineage cells) have been demonstrated during the course of NDD (Thome et al., 2018; Lunnon et al., 2012). Although both microglia and macrophages have been detected in CSF (Esaulova et al., 2020) and could potentially be isolated and characterized, so far no studies exist utilizing their presence in CSF in order to study the subset of microglia and macrophages in NDD. It has been demonstrated that the initiation and termination of the neuroinflammatory response of these cells often relies on epigenetic changes (Kaminska et al., 2016), and that the cause of their dysregulation is also often caused by epigenetic factors (Martins-Ferreira et al., 2021). Interestingly focusing on the epigenetics of these cells may provide clues about the state of both their plasticity and reveal various NDD specific microglial and peripheral leukocyte phenotypes (Cheray and Joseph, 2018; Wang et al., 2021; Masliah et al., 2013).

### 3.2 Imaging

Imaging has also been employed in the detection of myeloid biomarkers. Most widely known is the use of imaging for the detection of microglia- and macrophage-related neuroinflammation in the brain, by detecting the translocator protein (TSPO) with the help of radioligands (such as [^11^C]PK11195, [^18^F]DPA-714) and positron emission tomography (PET) (Zhou et al., 2021). A systematic review showed that there are increases in specific brain regions in a variety of inflammatory brain disorders, as well as in NDD (De Picker et al., 2023). A limitation is that other CNS-resident cells, such as astrocytes, can also express TSPO, and that microglia overactivation cannot easily be distinguished from gliosis in the scans. There are studies indicating that TSPO imaging indeed may reveal microglial activation but it is hard to determine whether this is protective or detrimental (Beckers et al., 2018). Others argue that while this type of imaging may be useful in rodents in detecting microglial activation/reactivity or gliosis, in humans the sensitivity is suboptimal for this (Nutma et al., 2023). Other methods, such as Magnetic resonance imaging (MRI) with for example susceptibility-weighted imaging (SWI) (Stüber et al., 2016) or quantitative susceptibility mapping (QSM) (Kaunzner et al., 2019), are used to detect iron deposition in CNS myeloid cells which is associated with NDD (Andersen et al., 2014). Additionally, Single-photon emission computed tomography (SPECT) (Jiemy et al., 2018) has also shown promise as a method to measure activation of myeloid cells in the brain.

**Figure 1:**
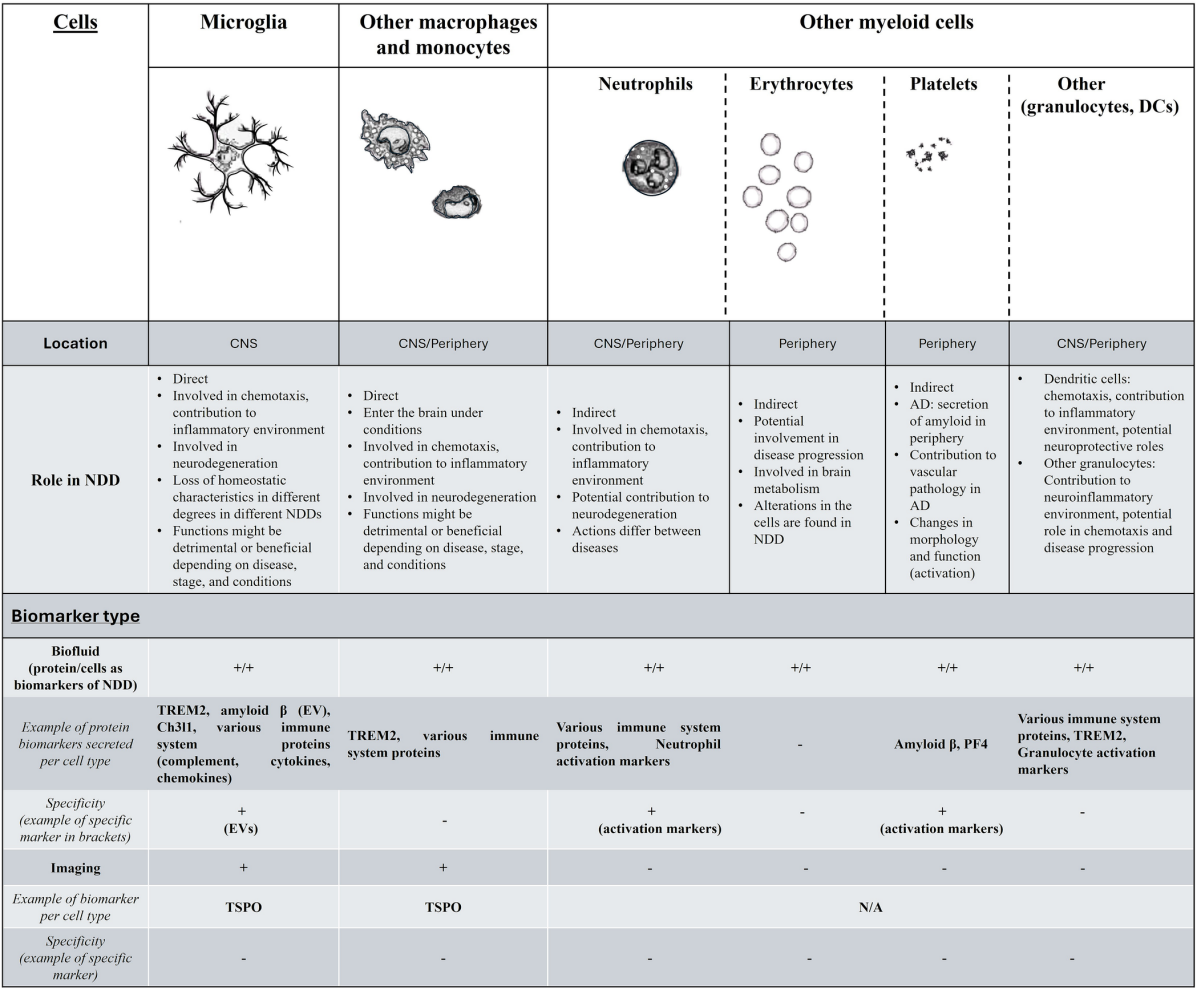
A summary of the findings for myeloid cells and biomarkers in neurodegenerative disorders. In the first part of this figure/table we include the myeloid cells that have been relevant to NDD, their location, and function in NDDs. For the second part (Biomarker type) while biofluid markers exist and are easy to research for monitoring the function of all myeloid cells, these are not cell specific (with the exception of markers such as neutrophil- or platelet-specific activation markers or EVs), while other biomarkers ranging from genetics and epigenetics-related, or cell behavior and morphology are more widely accessible, but under-investigated. The existence of biofluid biomarker per cell type is indicated with + for either proteins secreted that have been found to have potential as biomarkers in NDD (with examples) or for cell-specific changes that have been observed that can also act as biomarkers (behavior, function, morphology etc.). Myeloid cell related imaging biomarkers for NDD are currently limited to macrophage and microglia-specific, without the ability to distinguish between the two. More details are found in the text. DC, dendritic cells; NDD, neurodegenerative disorders; EV, extracellular vesicles.

The summary of the current findings for the above are demonstrated in Figure 1.

## 4 Discussion: challenges, gaps, and future of myeloid cells and biomarkers research

Developing markers that are more specific to a cell type or even a tissue (CNS vs. periphery derived biomarkers) would improve the precision of diagnostics and research. For myeloid cells, due to their ubiquity throughout the body, isolating and focusing on CNS findings is rather challenging. Although there are clearly encouraging results from CSF biomarkers, and both blood and imaging studies also show promising findings there is a need for improvement in sensitivity, specificity, and functional correlation, while taking into account that NDD processes change during the disease. For example, the detection of activated microglia, may signify the early stages of NDD, or could be due to other non-NDD etiology. Further, in the development of AD microglia get over-activated and hyper-reactive (which may be referred as primed), and eventually become exhausted and present a senescent phenotype; currently there are no validated markers to distinguish between microglial states. Moreover, hyperactivation and senescence in microglia is also found in normal aging (Niraula et al., 2017), as well as non-microglial cells (Munawara et al., 2021; Sharma, 2021), which makes investigating this function using biomarkers rather complex, although there is potential of pharmaceutically targeting senescent cells (Chaib et al., 2022). Current research focusing on EVs from different CNS myeloid cells for example is promising, and so is the discovery of tissue-specific isoforms; both tissue and even cell-specific isoforms of proteins have been discussed as potential therapeutic targets elsewhere (Kjer-Hansen et al., 2024), and while there is progress in CNS-disease linked myeloid biomarker isoforms [e.g., the brain-specific complement system clusterin protein with both cell-type specific and subcellular isoforms (Herring et al., 2019), the brain-specific TREM2 isoform (Shaw et al., 2022), and brain region specific APP isoforms in mice (Löffler and Huber, 1992)] this field needs further studies.

Moreover, standardization of biomarker measurements is needed in both the components of the methods (e.g., method characteristics, such as antibodies, buffers, and technologies) and the characteristics of samples and the individuals they originate from. For example, a lot of protein levels and cell functions are subjects to circadian changes (Fonken et al., 2015; Fagiani et al., 2022), so studies without standardized collection protocols when it comes to when these samples were collected (either time of the day or time after waking up) will introduce unnecessary “noise” in the data. Perhaps more accurate record keeping for all factors that have been identified including time since waking up, and whether the sample donor has a normal sleep/awake routine could be used to address this and these factors could be used as covariates. Similarly differences in disease activity or disease type for diseases such as MS (Graber and Dhib-Jalbut, 2011) may lead to different results depending on the disease stage, so an overall MS group heterogeneity will be present that will make interpretation of any results difficult. Confounding factors such as pre-analytical conditions when it comes to studies from multi center sample collections should also be considered, ranging from consumables used for sample collection, to sample pre-processing storage temperatures and time, to post-processing storage conditions, as they have all been shown to affect a variety of analytes in different ways (Walter et al., 2020; Ruiz-Godoy et al., 2019). Lastly, investigating usual confounding factors in the context of the disease in focus and how that interaction may affect biomarkers differently (i.e., different disease biomarker signatures in males and females) should be taken into account. Especially in diseases where the immune system is involved it has been repeatedly shown that male and female immune systems are not the same, and that is reflected for example on the differential manifestation of neuroimmune (including ND) diseases in males and females (Alvarez-Sanchez and Dunn, 2023; Ferretti et al., 2018; Gillies et al., 2014). Moreover, even if we look at cell-specific functions of immune cells such as microglia there are differences in function and physiology (Yanguas-Casás, 2020; Kodama and Gan, 2019). A variety of reasons may be responsible for these changes, including the fact that the X chromosome contains a large amount of immune system related genes (Bianchi et al., 2012), and the differential exposure to factors such as hormones during development (Calvo and Einstein, 2023), but as NDDs manifest in different ways between sexes, we cannot expect for the markers of the disease or even treatment to be measured in the population as a whole. Although using statistics to account for sex as a confounding factor is an approach that is generally considered as appropriate, a suggestion for future studies would be a sex-specific characterization of the disease in question (including markers of development, diagnosis, and response to treatment) in addition to results in the population as a whole. Additionally, statistical methods of clustering patients in groups based on biomarkers depending on their function (e.g., neutrophil function, overall myeloid function etc.) may offer new ways of stratification of -already heterogenous- NDD and a divide and conquer approach may be more beneficial in both research and treatment.

The NDD myeloid biomarkers do tell us that there is often enhanced inflammation in NDD, but focusing on single-cell or single-process groups may tell a different story; for example, microglia have been found to be senescent in AD (Saez-Atienzar and Masliah, 2020; Hu et al., 2021) but also found to be primed (Bivona et al., 2023). This priming is an overall NDD phenomenon too (Perry and Holmes, 2014); both phenotypes are inflammatory but aid in the inflammation in different ways. When considering the regional and temporal heterogeneity of microglia (Tan et al., 2020) and other cell types, alongside factors such as genetics, epigenetics, and patient-specific variables, it becomes clear that simplistic approaches—such as targeting a single biomarker to treat neurodegenerative diseases (NDDs) as a whole—are often ineffective and potentially hazardous. A pertinent example is the recent development and approval of disease-modifying therapies (DMTs) for Alzheimer’s disease (AD), specifically anti-amyloid drugs (Mintun et al., 2021). While some individuals experience delayed progression in certain aspects of the disease and a reduction in a myeloid cell-related biomarker (amyloid plaques), these treatments have been associated with severe side effects, such as amyloid-related imaging abnormalities (ARIA) (Roytman et al., 2023), which remain unpredictable in their manifestation and potentially lethal. These adverse effects stem from both vascular and immune system dysfunctions, with evidence—at least from mouse models (Taylor et al., 2023)—indicating involvement of myeloid cells. Therefore, caution is essential when prioritizing a single biomarker, as it may interact with and influence the broader biological system in unforeseen ways.

Similarly, ignoring resilience to the development of symptoms, or delay of pathology development regardless of the presence of the factors that otherwise indicate disease progression, misses opportunities to learn more about mechanisms which may lead to treatments. An unorthodox way to show this is by mentioning a recent study that indicated that the mechanisms that aid in developing neuroinflammation in MS, may inhibit the development of amyloidogenesis in AD (Brier et al., 2024), although tau may be telling a different story in the same disease (Zeydan et al., 2020). This could indicate that the mechanisms responsible for the development of MS are inhibitory toward amyloid pathology, or the drugs used in MS (as some participants of both studies were on MS DMT) had that effect, both of which could be investigated by using myeloid biomarkers. Conversely, conditions such as depression (Schneider et al., 2011), traumatic brain injury (Brett et al., 2022), and viral infections (Levine et al., 2023) have been shown to be associated with the future development of NDD so studying the relationship between these conditions and NDD development may lead to early stage NDD markers. Looking at both resilience factors and risk factors and how these interact, may lead to further stratification of NDD to more functional categories than only phenotype, which will potentially make a difference in how we think of and treat these diseases.

Focusing on cell-based studies, in this category characteristics are altered both under normal conditions, and upon activation of the cells *in vitro,* depending on the study. This indicates that the immune system dysfunction present in NDD (or pre-NDD) may -in cases- only appear upon activation of the immune system. Similarly, with the differences in disease activity discussed in the context of MS, differences in immune response may be subject to immune system triggering and activation.

It is apparent that when considering myeloid cells and biomarkers and their relationship with NDD, we need to not only think and study beyond microglia, but often beyond the CNS. Instead of having a disease focus in biomarker research, we need to understand what the variations within and between diseases mean, and how they translate to function and dysfunction, as the brain’s immune privilege disappears when the nature of the disease is (neuro)immune.
